# BMP-7 Attenuates Sarcopenia and Adverse Muscle Remodeling in Diabetic Mice via Alleviation of Lipids, Inflammation, HMGB1, and Pyroptosis

**DOI:** 10.3390/antiox12020331

**Published:** 2023-01-31

**Authors:** Chandrakala Aluganti Narasimhulu, Dinender K. Singla

**Affiliations:** Division of Metabolic and Cardiovascular Sciences, Burnett School of Biomedical Sciences, College of Medicine, University of Central Florida, Orlando, FL 32816, USA

**Keywords:** cell death, diabetic myopathy, inflammation, inflammasome, muscle atrophy

## Abstract

Diabetic myopathy involves hyperglycemia, oxidative stress, and inflammation. However, the role of hypercholesterolemia-induced inflammation-mediated pathological mechanisms leading to fibrosis, sarcopenia, deterioration of muscle, and muscle dysfunction in diabetes is not well understood. In this study, we investigated the novel role of bone morphogenetic protein-7 (BMP-7) in ameliorating metabolic alterations, inflammation, pyroptosis, TGF-β/SMAD cell signaling mechanisms, and progression of diabetic myopathy. C57BL/6J mice were treated with saline, streptozotocin (STZ), or STZ+BMP-7. Diabetes was confirmed by increased fasting glucose levels and a glucose tolerance test. Gastrocnemius muscle and blood samples were collected for lipid and tissue analysis using various methods. A significant increase in hyperglycemia resulted in an increase in lipid accumulation, monocyte infiltration, and inflammation, as well as an increase in pyroptotic markers and signaling markers in diabetic muscle myocytes. A structural analysis showed significant muscle loss, and increased muscle deterioration and fibrosis leading to muscle dysfunction. BMP-7 attenuated pathological processes that resulted in significantly improved muscle function. We report, for the first time, that increased hyperlipidemia aggravates inflammation-induced pyroptosis, resulting in significant muscle loss, sarcopenia, and adverse skeletal muscle remodeling in diabetic muscle myopathy. Interventional treatment with BMP-7 attenuates hypercholesterolemia-induced inflammation-mediated sarcopenia and adverse muscle remodeling, suggesting BMP-7 could be a potential treatment option for diabetic muscle myopathy.

## 1. Introduction

Sarcopenia is the loss of active skeletal muscle mass and function that reduces quality of life in aged people and individuals suffering from a variety of chronic diseases [1]. Sarcopenia has also been observed in rodents, monkeys, and humans with cardiovascular disease or type 2 diabetes [2,3]. Diabetes is associated with increased hyperglycemia, oxidative stress, and inflammation [4,5]. Recent reports have suggested that hyperglycemia led to muscle atrophy and remodeling, worsening to sarcopenia and muscle deterioration in frank diabetes [4,6]. Sarcopenia in patients with diabetes is of increasing concern given that the condition is associated with severity of muscular and cardiovascular events, and increased mortality [7].

Complexity in the development and progression of diabetes makes it difficult to establish causality or the trigger of diabetic sarcopenia, though some have suggested hyperglycemia-induced oxidative stress may be the initiating factor [8,9]. Hyperglycemia and oxidative stress often result in cell deaths, such as apoptosis, necrosis, and pyroptosis [10,11]. Apoptosis is a regulated form of programmed cell death, whereas necrosis is passive accidental cell death. Pyroptosis is an inflammation-mediated cell death reported to occur in various cardiovascular, metabolic, and skeletal muscle diseases [11,12]. However, the exact causes of inflammation, inflammation-induced pyroptosis, and sarcopenia in animals and humans is still debated. Moreover, whether increased hyperglycemia leads to lipid accumulation and functional alterations in diabetic muscle, or whether hyperglycemia is related to inflammation or inflammation-induced pyroptosis, is unknown. Diabetic sarcopenia and associated atrophy lead to adverse skeletal muscle remodeling which could be mediated through complex cell signaling mechanisms involving metabolic alterations and monocyte infiltrations of tissue. Further, the altered diabetic metabolic responses may lead to infiltration of fatty acids into the muscle, which could be a triggering agent in inflammation-induced sarcopenia and muscle remodeling.

Several pharmacological agents, including glucose lowering drugs (metformin, insulin, sulfonylureas, dipeptidyl peptidase-4 (DPP4) inhibitors, thiazolidinediones, sodium-glucose cotransporter 2 (SGLT2) inhibitors, and glucagon-like peptide-1 receptor agonists), hormones (testosterone), antioxidants (vitamins-C, D, E, and N-acetylcysteine), and nonpharmacological therapies such as resistance training, and protein and amino acid supplementation, have been used to treat diabetic sarcopenia [13,14]. However, the cost and side effect profile of many of these therapies has limited their use [15,16]. Additionally, except for metformin, anti-hyperglycemic drugs affect only glucose control with no effect on cholesterol metabolism. Clinical evidence suggests metformin reduces serum low-density lipoprotein cholesterol (LDL-C) level effectively [17,18], but its use is limited due to adverse side effects [19]. Hence, there is a need for alternative strategies to attenuate diabetic hyperlipidemia-associated sarcopenia and adverse cardiac remodeling. Recently, we identified that bone morphogenetic protein-7 (BMP-7) reduced hyperglycemia and sarcopenia in diabetes [11], but whether BMP-7 could reduce diabetic cholesterol levels, potentially linked with inflammation, sarcopenia and adverse muscle remodeling, has not yet been addressed. 

The objectives of this study were: (1) to understand the role of blood cholesterol, cholesterol-loading genes, and tissue accumulation of lipids leading to monocyte infiltration, inflammation, inflammation-induced cell death, muscle atrophy, adverse muscle remodeling, and sarcopenia, (2) to understand the involvement of proinflammatory and anti-inflammatory cell signaling pathways in the context of diabetic muscle myopathy and sarcopenia, and (3) to understand whether treatment with BMP-7 can attenuate cholesterol levels and tissue lipid accumulation, inflammation, pyroptosis, sarcopenia, and muscle dysfunction.

## 2. Materials and Methods

### 2.1. Experimental Design

**Figure 1:**
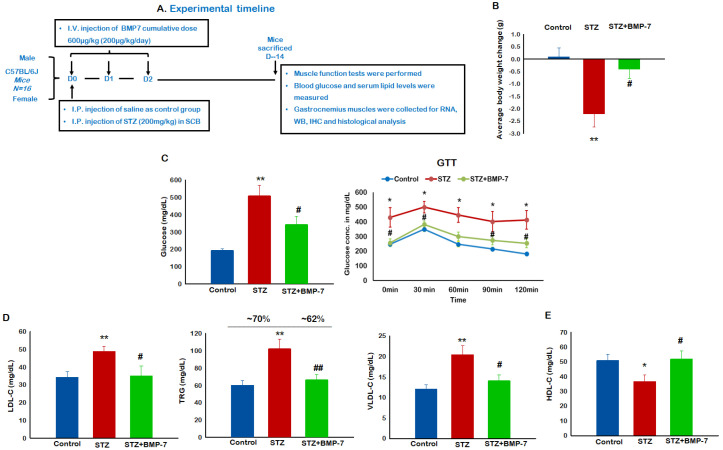
(**A**) Effects of BMP-7 treatment on diabetes-induced weight loss, hyperglycemia, and systemic hypercholesterolemia. Experimental timeline, at Day 14 after STZ administration mice body weight, glucose levels, and blood lipids were measured; (**C**,**D**) bar graphs represent elevated glucose levels (**C**) and lipid levels including LDL, TRG, and VLDL (**D**) of STZ-administered mice, whereas BMP-7 treatment potentially reduced the glucose and lipid levels on D-14, and line graph (**C**) represents GTT; (**B**,**E**) in addition, BMP-7 improved the HDL cholesterol levels in diabetic mice (**E**) and improved the diabetes-induced weight loss (**B**). Error bars = mean ± standard error of the mean. One-way ANOVA and Tukey tests were performed to assess statistical significance. * *p* < 0.05, ** *p* < 0.01 vs. control; # *p* < 0.05, ## *p* < 0.01 vs. STZ; *n* = 12–16; *n*, number of animals.

All animal procedures in the current study were performed according to approval by the Institutional Animal Care and Use Committee (IACUC) of the University of Central Florida and according to National Institutes of Health (NIH) guidelines. A total of 48 C57BL/6J male and female mice (Jackson labs, Bar Harbor, ME, USA) of 10 ± 2 weeks age, weighing 20–25 g, were used (Figure 1A). Mice were divided into three groups and BMP-7 injections were administered, as described previously [11]. Body weights were measured both prior to streptozotocin (STZ, MP Biomedicals, Irvine, CA, USA) injection and at the end of the study. On Day 14 (D-14), muscle function was tested. Blood samples were collected by heart puncture via exsanguination, and animals were euthanized under 4% isoflurane followed by cervical dislocation. Gastrocnemius (GM) muscles were collected, washed with phosphate-buffered saline (PBS), weighed, and stored either at −80 °C in RNAlater (Thermo Fisher, Waltham, MA, USA) for gene expression and Western blotting or in 4% paraformaldehyde (PFA, Thermo Fisher) for histological staining.

### 2.2. Blood Glucose Measurements

Blood pricks were conducted to measure blood glucose levels at D-14 using a OneTouch Ultra Mini glucose meter (LifeScan, Milpitas, CA, USA), as described previously [11,20].

### 2.3. Glucose Tolerance Test (GTT)

Fourteen days after STZ administration, following measurement of fasting blood glucose levels, control and experimental animals were subjected to GTT [20]. In brief, mice were fasted overnight, and initial blood glucose levels were measured. Glucose (1 g/kg body weight, Sigma, St. Louis, MO, USA) was administered intraperitoneally, and blood glucose levels were measured via tail vein puncture every 30 min until 120 min.

### 2.4. Blood Lipid Quantification

Serum lipid profile was determined using a Cholestech L*D*X analyzer (Cholestech, Hayward, CA, USA), following the manufacturer’s instructions [21,22,23]. Briefly, 45 µL of serum sample was used to analyze lipid levels in all groups.

### 2.5. Tissue Processing

Formalin-fixed muscle tissues were rinsed three times with PBS and stored in 70% ethanol until processing. Different percentages of ethanol solutions were used to dehydrate the GM, followed by CitriSolv (Decon Laboratories Inc., King of Prussia, PA, USA) and tissue processing using a TP1020 automatic benchtop tissue processor (Leica, Allendale, NJ, USA). Muscle tissues were embedded in paraffin wax using a Tissue-Tek TEC 6 (Sakura Finetek, Torrance, CA, USA). Paraffin-embedded tissues were cut into 5 μm sections and placed onto Colorfrost Plus slides for histological analysis.

### 2.6. Histological Staining

IHC staining was performed as published previously [24,25]. GM sections were deparaffinized followed by rehydration. Sections were blocked with 10% normal goat serum (NGS, Vector Laboratories, Burlingame, CA, USA). Following blocking, sections were stained (Table S1A) for myosin, high mobility group box 1 (HMGB1), toll-like receptor 4 (TLR-4), NOD-, LRR-, and pyrin domain-containing protein 3 (NLRP3), caspase-1, interleukin-1 beta (IL-1β), IL-18, gasdermin D (GSDMD), perilipin, CD14, and inducible nitric oxide synthase (iNOS). Secondary antibodies Alexa Fluor^®^ 488 or 568 goat anti-rabbit (Invitrogen, Carlsbad, CA, USA) were used. For perilipin/laminin staining, a second blocking step was performed prior to co-staining with laminin to identify the muscle fiber border. Finally, sections were washed, and nuclei were counterstained with mounting medium containing DAPI (4′,6-diamidino-2-phenylindole, #H-1200, Vector Laboratories).

To determine the interstitial and vascular fibrosis, Masson’s trichrome staining was performed and quantified as described previously [11,20,26,27,28]. To evaluate myofibrillar loss, GM sections were stained with hematoxylin and eosin, and muscle myocyte size (mm^2^) was quantified as described previously [11,27,29]. All images were taken with a Keyence microscope (BZ-X810-Keyence, Itasca, IL, USA), 20X images were analyzed in ImageJ 1.39o (Bethesda, MD, USA), and representative, enlarged images were taken at 40X magnification.

### 2.7. Real-Time Polymerase Chain Reaction (RT-PCR)

RNA was isolated from GM tissue, cDNA synthesized (SuperScript^®^III First-Strand Synthesis Super Mix kit, Thermo Fisher), and quantitative real-time PCR was performed for atrogin-1, glyceraldehyde-3-phosphate dehydrogenase (GAPDH), caspase-1, carnitine palmitoyltransferase 1 (CPT1), fatty acid binding protein 1 (FABP1), pyruvate dehydrogenase kinase 4 (PDK4), fibroblast growth factor 21 (FGF21), GSDMD, HMGB1, IL-1β, IL-18, matrix metalloproteinase 9 (MMP9), muscle ring finger 1 (MuRF1), NLRP3, TLR4, CD36, scavenger receptor A1 (SR-A1), ATP binding cassette subfamily A member 1 (ABCA1), ATP binding cassette subfamily G member 1 (ABCG1), CD14, inducible nitric oxide synthase (iNOS), toll-like receptor (TLR-4), TGF-β1, SMAD family member 2 (Smad2), Smad3, and perilipin (Table S1B). Fold expression was calculated using the 2^−∆∆Ct^ method and normalized to GAPDH, as described previously [11].

### 2.8. Western Blotting

GM tissue (15–20 mg) was homogenized using radioimmunoprecipitation (RIPA) lysis buffer, supernatant was collected after centrifugation, and protein concentration was estimated using a Bio-Rad protein assay (Hercules, CA, USA). Protein samples (25 µg) were loaded and run on Bolt gels (10–15%, Thermo Fisher) for 22 min at 200 V. The gels were transblotted onto iBlot 2 transfer kits using an iBlot 2 Western blot transfer system (Invitrogen, Carlsbad, CA, USA) for 7 min. Membranes were blocked with either 5% non-fat milk or bovine serum albumin (BSA) in 1X TBST (tris-buffered saline, 0.1% Tween-20) at room temperature (RT) for 1h, then incubated with primary antibodies (Table S1A) for TGF-β1, phosphorylated SMAD2 (pSMAD2), pSMAD3, pSMAD1/5/9, and GAPDH overnight at 4 °C. Following primary antibody incubation, membranes were washed with 1X TBST and incubated with horseradish peroxidase-conjugated goat anti-rabbit IgG secondary antibody for 1 h at RT. Finally, membranes were exposed to enhanced chemiluminescence (ECL, Thermo Fisher) reagent and the signal was detected using X-ray films. Densitometric analysis was performed using the ImageJ software on scanned X-ray films. All protein band intensities were normalized to GAPDH and expressed as arbitrary units (a.u.).

### 2.9. Muscle Strength Tests

Muscle function was assessed using a grip strength meter [30,31], rotarod [32,33], and weights tests [34] on D-14. Grip strength was performed as described previously [11]. Muscle endurance was assessed via rotarod as described previously [11]. Muscle strength was assessed by performing the weights test procedure as reported previously [11,34]. Time to hold weight in seconds was recorded. Normalized forelimb muscle strength was calculated.

### 2.10. Cytokine Array

The cytokine expression profiles of GMs from all the three groups were analyzed using a RayBio^®^ C-Series Mouse Cytokine Antibody Array 1000 (#AAM-CYT-1000, RayBiotech, Peachtree Corners, GA, USA), following the manufacturer’s instructions. Images were recorded and a densitometric analysis was performed using ImageJ; values were normalized to the control for diabetic samples and to diabetic samples for BMP-7 treatment samples.

### 2.11. Statistical Analysis

Values are presented as mean ± standard error of the mean (SEM). Statistical significance was assessed between groups via Student’s *t*-test and among groups via one-way analysis of variance (ANOVA), followed by Tukey’s post hoc test, using the statistical software Sigma Plot (versions 10 and 12.5, Systat, San Jose, CA, USA), with *p* < 0.05 considered to be statistically significant.

## 3. Results

### 3.1. Effects of BMP-7 Treatment on Diabetes-Induced Weight Loss

We observed clear, statistically significant (*p* < 0.05) differences in body weight between the control and diabetic mice (Figure 1B). Following the BMP-7 treatment, diabetic mice showed a significant increase (*p* < 0.05, ~82%) in body weight (Figure 1B), suggesting BMP-7 treatment attenuates weight loss with the potential to improve metabolism in the setting of diabetes. 

### 3.2. Effects of BMP-7 Treatment on Diabetic Hyperglycemia

Blood glucose levels were significantly (*p* < 0.05, ~1.62-fold) elevated in diabetic mice as compared with the control mice, whereas the BMP-7 treatment significantly (*p* < 0.05, ~74%) attenuated diabetes-induced hyperglycemia (Figure 1C). The GTT test revealed blood glucose levels, and the incremental glucose curve over time (60–120 min) was significantly reduced in the BMP-7 group as compared with untreated diabetes, suggesting BMP-7 treatment significantly ameliorated hyperglycemia (*p* < 0.05, Figure 1C) in diabetic mice. 

### 3.3. Effects of BMP-7 Treatment on Diabetic Systemic Hypercholesterolemia

A significant (*p* < 0.05) increase in the levels of serum low-density lipoprotein cholesterol (LDL-C, (~42%), triglycerides (TRG, ~72%), and very low-density lipoprotein cholesterol (VLDL-C, ~70%) was observed in diabetic mice as compared with the control mice (Figure 1D). Interestingly, a significant (*p* < 0.05, Figure 1E) decrease in high-density lipoprotein cholesterol (HDL-C, ~28%) levels was observed in diabetic mice, whereas BMP-7 treatment significantly (*p* < 0.05) attenuated diabetes-induced LDL-C (~28%), TRG (~62%), and VLDL-C (~59%) increases. Importantly, our data indicated a significant (*p* < 0.05, ~95%) increase in HDL-C levels with BMP-7 treatment.

### 3.4. BMP-7 Treatment Reduces Diabetic Lipid Accumulation in Skeletal Muscle

**Figure 2:**
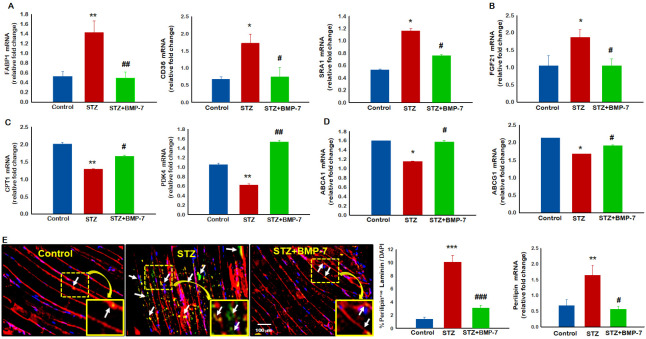
BMP-7 treatment reduces diabetes-induced lipid accumulation in muscle. At Day 14 after STZ administration, mice GM tissues were evaluated for tissue lipid accumulation by RT-PCR and IHC staining using laminin (Red) and perilipin. Histograms represent (*n* = 5–6) BMP-7 reduced systemic hypercholesterolemia and (**A**) cholesterol loading genes FABP1, CD36, and SRA1; (**B**) stress-induced myokine FGF21; increased (**C**) fatty acid oxidation genes CPT1 and PDK4; and improved the reverse cholesterol transport genes (**D**) ABCA1 and ABCG1. As shown in panel (**E**), lipid accumulation (Perilipin-green) significantly increased in diabetic (STZ) mice as compared with the control mice. BMP-7-treated diabetic mice had significantly lesser accumulation of perilipin. White arrows indicate the lipid accumulation. Bar graphs represent quantitative analysis for IHC and perilipin gene expressions. Error bars = mean ± standard error of the mean. One-way ANOVA and Tukey tests were performed to assess statistical significance. * *p* < 0.05, ** *p* < 0.01, *** *p* < 0.001 vs. control; # *p* < 0.05, ## *p* < 0.01, ### *p* < 0.001 vs. STZ; (*n* = 6–8); *n*, number of animals. FABP1, fatty acid binding protein 1; SR-A1, scavenger receptor A1; FGF21, fibroblast growth factor 21; CPT1, carnitine palmitoyltransferase 1; PDK4, pyruvate dehydrogenase kinase 4; ABCA1, ATP binding cassette subfamily A member 1; ABCG1, ATP binding cassette subfamily G member 1.

Next, we analyzed gene expressions for cholesterol loading genes FABP1, CD36, and SR-A1. A significant (*p* < 0.05) increase in cholesterol loading gene expression (Figure 2A) was observed in diabetic mice GM as compared with the control mice, whereas a significant (*p* < 0.05) reduction was observed with BMP-7 treatment. Moreover, expression of a specific stress-induced myokine, FGF21, was significantly (*p* < 0.05 Figure 2B) increased in the GM of diabetic mice. Following the BMP-7 treatment, we observed a significant reduction in FGF21 expression. To further understand the involvement of hypercholesterolemia in diabetes, we measured expression of genes, CPT1, and PDK4, related to fatty acid oxidation. Our data showed CPT1 and PDK4 (Figure 2C) genes were significantly (*p* < 0.05) reduced in diabetic mice as compared with the control mice. A significant upregulation of these genes was observed with the BMP-7 treatment. Further strengthening our data, we evaluated the expression of genes inherent to RCT, ABCA1, and ABCG1. A significant (*p* < 0.05) decrease in RCT-specific genes (Figure 2D) was observed in diabetic mice as compared with the control mice, whereas a significant (*p* < 0.05) increase was observed with the BMP-7 treatment.

To determine whether an increase in cholesterol-specific genes in diabetic GM led to tissue infiltration of lipids and cellular dysfunction, we performed perilipin immunostaining. Our data showed significantly (*p* < 0.05, Figure 2E) increased lipid accumulation in diabetic skeletal mice as compared with the control mice. A significant reduction in fat deposition was observed upon BMP-7 treatment. Quantitative data for fat deposition were significantly increased in diabetic mice as compared with the control mice, whereas BMP-7 treatment significantly (*p* < 0.05, Figure 2E) reduced fat deposition in diabetic mice. Additionally, in some areas of muscle, perilipin staining appeared to penetrate discontinuous laminin borders of muscle fibers, suggesting direct replacement of degenerating muscle. To further confirm our IHC results, RT-PCR analysis was performed for perilipin gene expression which was consistent with IHC data. A significant (*p* < 0.05) increase in perilipin gene expression (Figure 2E) was observed in diabetic mice as compared with the control mice, whereas a significant (*p* < 0.05) decrease was observed with the BMP-7 treatment. These data raised an interesting question as to whether enhanced systemic hypercholesterolemia and GM-specific lipid accumulation would lead to monocyte infiltration and inflammation.

### 3.5. Effects of BMP-7 Treatment on Monocyte Infiltration, M1-Macrophage Polarization, and Inflammation

**Figure 3:**
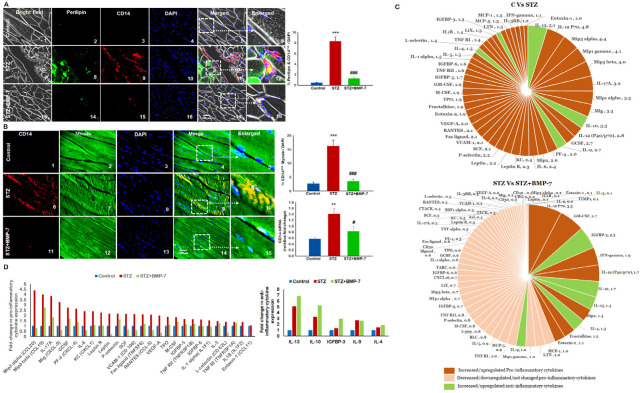
Effects of BMP-7 treatment on monocyte Infiltration and inflammation: (**A**) Represents monocyte infiltration near lipid accumulation. As shown in figure bright field images (1, 7, 13), perilipin-positive cells in green (2, 8, 14); CD14 stained in red (3, 9, 15); total nuclei stained with DAPI in blue (4, 10, 16); and merged images (5, 11, 17). Dotted white boxes and arrows indicates enlarged sections of merged images (6, 12, 18) demonstrates colocalization of CD14, perilipin, and DAPI. Scale bar = 100 μm. Error bars = mean ± standard error of the mean. One-way ANOVA and Tukey tests were performed to assess statistical significance. Bar graph represents quantitative analysis for IHC (*n* = 6–8). ** *p* < 0.001 vs. control; ### *p* < 0.001 vs. STZ; *n*, number of animals. (**B**) Hyperlipidemia-induced oxidative stress causes monocyte infiltration-mediated inflammation which furthers cellular pathological mechanisms. Representative images of IHC staining for CD14 expression at D14 shows that administration of STZ significantly increases CD14 expression in STZ mice (6~10) as compared with the control mice (1~5). BMP-7-treated diabetic mice had significantly lower number of +ve cells (11~15. In all figures, CD14 marker is shown in red (1, 6, 11), muscle cells in green (2, 7, 12j), DAPI in blue (3, 8, 13), and merged images (4, 9, 14). Scale bar = 100 μm. White dotted boxes and arrows indicate enlarged sections of merged images (5, 10, 15). Quantitative analysis in bar graph for IHC (*n* = 6–8); gene expression (*n* = 5–6) shows increased expression of CD14 in GM of STZ mice vs. control mice, whereas BMP-7 treatment showed significant reduction of CD14 in diabetic mice. Error bars = mean ± standard error of the mean. One-way ANOVA and Tukey tests were performed to assess statistical significance. ** *p* < 0.01, *** *p* < 0.001 vs. control; # *p* < 0.05, vs. STZ; *n*, number of animals; iNOS, inducible nitric oxide synthase. (**C**) Cytokine array data showed 46 cytokines increased in the diabetic group as compared with the control (proinflammatory, dark brown and anti-inflammatory, light green). We stratified cytokines into 5 categories based on fold-change expression, with categories 1–4 representing proinflammatory cytokines, and category 5, anti-inflammatory. Cytokines elevated greater than 3-fold included IL-12p70, MIP3α, MIP1γ, MIP3β, IL-17A, MIP1α, and MIG. Category 2 consisted of 13 cytokines with a fold-change between 2 and 3, including IL-12p40/70, GCSF, PF4, MIP2, IL-6, KC, leptin R, P-selectin, SCF, VCAM1, FasL, and RANTES. Category 3 included 10 cytokines with a fold-change between 1.5 and 2, including eotaxin 2, fractalkine, TPO, M-CSF, GM-CSF, IGFBP-5, TNFRII, IGFBP6, IL-1α, and IL-5. Eleven cytokines in Category 4 had a fold-change between 1 and 1.5. Category 5 included anti-inflammatory cytokines, with variable fold-changes between 1.3 and 5.1, including IGFBP3, IL-4, IL-9, IL-10, and IL-13. Next, we repeated the analysis for untreated diabetic versus BMP-7-treated mice. The cytokine array data showed 62 cytokines upregulated or downregulated upon BMP-7 treatment. Cytokines were grouped again based on fold-change in expression, into 3 categories. Category 1 included cytokines with a fold-change of over 1.1, such as eotaxin 1, fractalkine, MIP2, IL-12p40/70, IFNγ, GM-CSF, and IL-12p70 (dark brown). Category 2 included 50 proinflammatory cytokines with reduced expression, or no change as compared with the diabetic group (light brown). Anti-inflammatory cytokines comprised Category 3, depict a 1.3- to 2.3-fold increase (light green). Diabetic conditions increased proinflammatory cytokine production, while BMP-7 treatment reduced proinflammatory, and increased anti-inflammatory, cytokine production. (**D**) A total of 96 proinflammatory cytokines were analyzed of which there was a ~2–5-fold increase in the diabetic sample as compared with the control sample. More specifically, we observed the proinflammatory MIP3α, MIP3β, IL-17A, MIG, GCSF, PF-4, IL-6, KC, leptin, p-selectin, SCF, VCAM-1, Fas ligand, RANTES, VEGF, TPO, MCSF, TNFRII, IL-1 α, IL-5, TNFRI, and IL-1 beta (ranging from ~1.5 to 4.5-fold) under diabetic condition as compared with the control sample, whereas BMP-7 treatment reduced differentially all the proinflammatory cytokine expression. BMP-7 treatment showed increased anti-inflammatory cytokines (IL-13, IL-10, IGFBP3, IL-9, and IL-4) as compared with other groups. FasL, Fas ligand; GCSF, granulocyte colony-stimulating factor; GM, gastrocnemius muscle; GM-CSF, granulocyte-macrophage colony-stimulating factor; IL, interleukin; IGFBP-5, insulin-like growth factor binding protein 5; KC, keratinocyte chemoattractant; M-CSF, macrophage colony-stimulating factor; MIP, macrophage inflammatory protein; MIG, monokine-induced gamma interferon; PF4, platelet factor 4; RANTES, regulated upon activation, normal T cell expressed; SCF, stem cell factor; TNFRII, tumor necrosis factor receptor II; TPO, thyroperoxidase; VCAM1, vascular cell adhesion molecule 1; VEGF, vascular endothelial growth factor.

To evaluate hyperglycemia-induced lipid accumulation leading to monocyte infiltration, we performed double immunohistochemistry for lipid accumulation staining using perilipin and monocyte infiltration using monocyte marker CD14 (Figure 3A). Our data showed significantly increased monocyte infiltration at the site of lipid deposition in GM of diabetic mice (Figure 3(A7~12)) as compared with the control mice (Figure 3(A1~6)), suggesting lipid deposition seems to be a major cause of monocyte infiltration and initiation of inflammation in diabetic muscle. Further, quantitative data analysis for co-stained perilipin and monocyte marker CD14 showed a significant (*p* < 0.05, Figure 3A) increase in the number of positive cells in the diabetic group as compared with the control group. Interestingly, a significantly (*p* < 0.05) smaller number of infiltrated monocytes were observed upon BMP-7 treatment (Figure 3(A13~18)), suggesting its ability in attenuation of lipid accumulation and monocyte infiltration.

Next, we evaluated the total number of positive cells for monocyte infiltration and their differentiation into proinflammatory M1 macrophages using monocyte marker CD14 for monocytes and iNOS for M1 macrophages, as reported previously [12]. Representative images and quantitative analysis data demonstrated the presence of a higher number of CD14^+^ (Figure 3B) and iNOS^+^ (Figure S1A) cells in the diabetic group (2~10f–j) as compared with the control group (1~5). However, BMP-7 treatment significantly (*p* < 0.05) reduced CD14^+^ and iNOS^+^ cell numbers (11~15). Additionally, a quantitative analysis showed a significant (*p* < 0.05, Figure 3B and Figure S1B) increase in the number of positive cells in diabetic animals, whereas a significant (*p* < 0.05) reduction was observed with BMP-7 treatment. To fortify our results, we performed a further RT-PCR analysis for CD14 and iNOS to confirm the IHC data. A significant (*p* < 0.05) increase in CD14 and iNOS gene expressions (Figure 3B and Figure S1C) was observed in diabetic mice, whereas a significant (*p* < 0.05) reduction was observed with BMP-7 treatment.

### 3.6. Effects of BMP-7 Treatment on Cytokine Release

To determine the lipid accumulation and monocyte infiltration leading to increased inflammation, and whether BMP-7 treatment attenuates diabetes-induced inflammation, we performed a cytokine antibody array consisting of 96 proinflammatory and anti-inflammatory proteins. The cytokine array data (Figure 3C) showed 46 cytokines increased in the diabetic group as compared with the control group (proinflammatory, dark brown and anti-inflammatory, light green). We stratified cytokines into five categories based on fold-change expression, with Categories 1–4 representing proinflammatory cytokines, and Category 5 representing anti-inflammatory. Cytokines that were elevated greater than 3-fold included IL-12p70, MIP3α, MIP1γ, MIP3β, IL-17A, MIP1α, and MIG, largely proinflammatory cytokines associated with macrophages. Category 2 consisted of 13 cytokines with fold-changes between 2 and 3, including IL-12p40/70, GCSF, PF4, MIP2, IL-6, KC, leptin R, P-selectin, SCF, VCAM1, FasL, and RANTES. Category 3 included 10 cytokines with fold-changes between 1.5 and 2, including eotaxin 2, fractalkine, TPO, M-CSF, GM-CSF, IGFBP-5, TNFRII, IGFBP6, IL-1α, and IL-5. Eleven cytokines in Category 4 had fold-changes between 1 and 1.5. Category 5 included anti-inflammatory cytokines, with variable fold-changes between 1.3 and 5.1, including IGFBP3, IL-4, IL-9, IL-10, and IL-13.

Next, we repeated the analysis for untreated diabetic versus BMP-7-treated mice. The cytokine array data (Figure 3C) showed 62 cytokines upregulated or downregulated upon BMP-7 treatment. Cytokines were grouped again, based on fold change in expression, into three categories: Category 1 included cytokines with fold-changes of over 1.1, such as eotaxin 1, fractalkine, MIP2, IL-12p40/70, interferon-gamma (IFNγ), GM-CSF, and IL-12p70 (dark brown); Category 2 included 50 proinflammatory cytokines with reduced expression or no change as compared with the diabetic group (light brown); anti-inflammatory cytokines comprised Category 3, with from 1.3 to 2.3-fold increases (light green). Diabetic conditions increased proinflammatory cytokine production, while BMP-7 treatment reduced proinflammatory, and increased anti-inflammatory, cytokine production. 

A total of 96 proinflammatory cytokines were analyzed of which there was a ~2–5-fold increases in the diabetic sample as compared with the control sample. More specifically, we observed increased proinflammatory cytokines macrophage inflammatory protein (MIP)3 alpha, MIP3 beta, IL-17A, MIG, GCSF, PF-4, IL-6, KC, leptin, p-selectin, SCF, VCAM-1, Fas ligand, RANTES, vascular endothelial growth factor (VEGF), TPO, MCSF, TNFRII, IL-1 alpha, IL-5, TNFRI, and IL-1 beta (ranging from ~1.5 to 4.5-fold) under diabetic condition (Figure 3D) as compared with the control, whereas BMP-7 treatment reduced differentially the proinflammatory cytokine expression. Further, BMP-7 treatment showed increased anti-inflammatory cytokines (IL-13, IL-10, IGFBP3, IL-9, and IL-4, (Figure 3D)) as compared with the other groups. 

### 3.7. Effects of BMP-7 Treatment on Inflammation-Induced Pyroptosis

**Figure 4:**
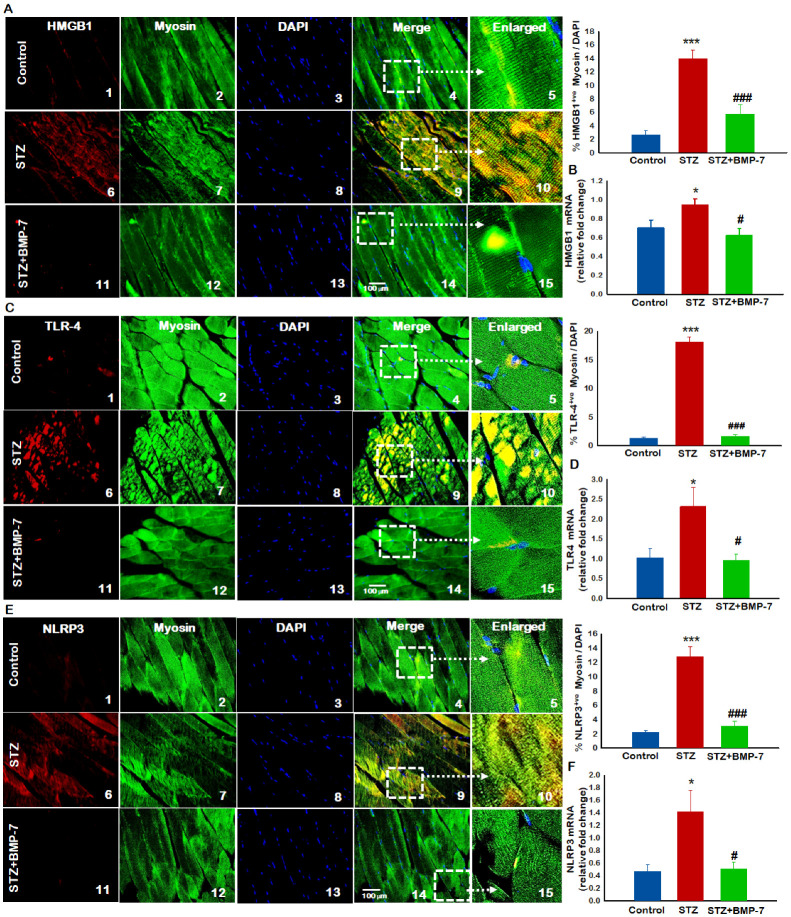
Effects of BMP-7 treatment on inflammation-induced pyroptosis hyperlipidemia-induced monocyte infiltration and inflammation triggers HMGB1, initiates the inflammasome activation and subsequent pyroptotic cascade results in pyroptotic cell death. Representative images of IHC staining for (**A**) HMGB1 (**C**) TLR4, and (**E**) NLRP3 expression at D14 show that administration to STZ significantly increases HMGB1, TLR4, and NLRP3 expression in diabetic mice (6~10) as compared with the control mice (1~5). BMP-7-treated diabetic mice had significantly lower number of +ve cells (11~15). In all figures HMGB1, TLR4 and NLRP3 markers showed in red (1, 6, 11), muscle cells in green (2, 7, 12j), DAPI in blue (3, 8, 13), and merged images (4, 9, 14). Scale bar = 100 μm. White boxes and arrows indicate enlarged sections of merged images (5, 10, 15). Quantitative analysis in bar graph for IHC (*n* = 6–8) gene expression (**B**,**D**,**F**) (*n* = 5–6) shows increased expression of HMGB1, TLR4, and NLRP3 in GM of STZ mice vs. control mice, whereas BMP-7 treatment showed significant reduction of all markers in diabetic mice. Error bars = mean ± standard error of the mean. One-way ANOVA and Tukey tests were performed to assess statistical significance. * *p* < 0.05 vs. *** *p* < 0.001 vs. control; # *p* < 0.05, ### *p* < 0.001 vs. STZ; *n*, number of animals; HMGB1, high mobility group box protein 1; TLR4, toll-like receptor; NLRP3, NOD-, LRR- and pyrin domain-containing protein 3.

To understand whether diabetic inflammation and infiltration of monocytes at the site of lipid deposition can lead to pyroptosis, we performed IHC for pyroptosis-specific initiators and components of the inflammasome, HMGB1, TLR-4, and NLRP3. Our data demonstrated the presence of a higher number of HMGB1^+^ (Figure 4A), TLR-4^+^ (Figure 4C), and NLRP3^+^ (Figure 4E) cells in the diabetic group (2~10) as compared with the control group (1~5). Moreover, BMP-7 treatment significantly (*p* < 0.05) reduced the number of positive HMGB1, TLR-4, and NLRP3 cells (11~15). Moreover, our quantitative data showed a significant (*p* < 0.05, Figure 4A,C) increase in the number of positive cells in diabetic animals, whereas a significant (*p* < 0.05) reduction was observed with BMP-7 treatment. To strengthen our findings, we performed an RT-PCR analysis for HMGB1, TLR-4, and NLRP3. Significant (*p* < 0.05) increases in HMGB1, TLR-4, and NLRP3 gene expressions (Figure 4B,D,F) were observed in diabetic mice as compared with the control mice, whereas a significant (*p* < 0.05) reduction was observed with BMP-7 treatment. Further gene expression analyses of HMGB1, TLR-4, and NLRP3 (Figure 4B,D,F) corroborated with the IHC data (Figure 4A,C,E).

**Figure 5:**
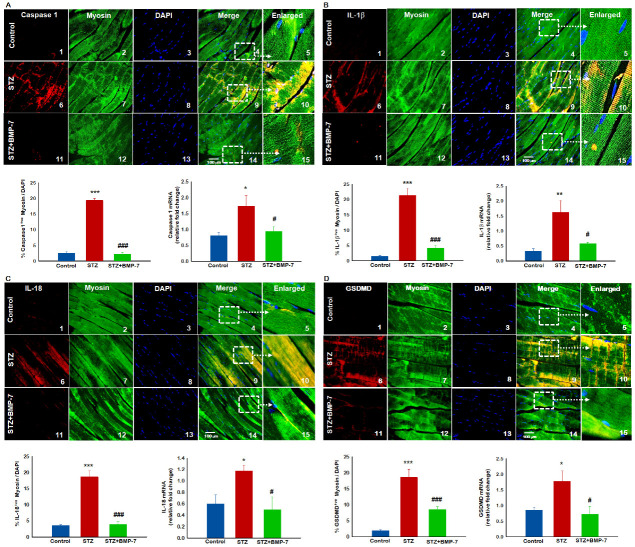
BMP-7 attenuates hyperlipidemia-induced pyroptotic cell death. HMGB1 initiated inflammasome mediates subsequent pyroptotic cascade which results in pyroptotic cell death. Representative images of IHC staining for pyroptosis markers (**A**) Caspase-1 (**B**) IL-1β (**C**) IL-18, and (**D**) GSDMD expression at D14 shows that administration of STZ significantly increases pyroptosis markers in STZ mice (6~10) as compared with the control mice (1~5). BMP-7-treated diabetic mice had significantly lower numbers of +ve cells (11~15). Positive cells for all markers are in red in each panel (1, 6, 11), muscle cells in green (2, 7, 12), DAPI in blue (3, 8, 13), and merged images (4, 9, 14). Scale bar = 100 μm. Dotted white boxes and arrows indicate enlarged sections of merged images (5, 10, 15). Quantitative analysis in bar graph for IHC (*n* = 6–8) gene expression (*n* = 5–7) shows increased expression for all markers in GM of STZ mice vs. control mice, whereas BMP-7 treatment showed significant reduction of all markers in diabetic mice. Error bars = mean ± standard error of the mean. One-way ANOVA and Tukey tests were performed to assess statistical significance. * *p* < 0.05, **p < 0.001, *** *p* < 0.001 vs. control; # *p* < 0.05, ### *p* < 0.001 vs. STZ; *n*, number of animals; IL, interleukin; GSDMD, gasdermin D.

Next, to determine whether pyroptosis initiator activation and inflammasome formation leads to formation of the pyroptosis cascade (caspase-1, and downstream markers IL-1β and IL-18), pyroptosis executioner, GSDMD [35], and cell death, we performed an IHC and gene expression analysis. Our data demonstrated significantly (*p* < 0.05) higher positive cells for pyroptosis markers caspase-1 (Figure 5A), IL-1β (Figure 5B), IL-18 (Figure 5C), and GSDMD (Figure 5D) in the diabetic group (6~10) as compared with control group (1~5). Diabetic mice treated with BMP-7 showed significantly (*p* < 0.05) reduced positive cells for pyroptosis-specific markers (11-15). Our pyroptotic cascade quantitative data showed a significant (*p* < 0.05, Figure 5A,D) increase in the number of positive cells in the diabetic group, and this increase was significantly (*p* < 0.05) reduced with BMP-7 treatment. To strengthen our results, we performed an RT-PCR analysis to confirm the IHC data. Significant (*p* < 0.05) upregulation of caspase-1, IL-1β, IL-18, and GSDMD gene expressions was observed in the diabetic (Figure 5A,D) group as compared with the control group, whereas BMP-7 treatment significantly downregulated caspase-1, IL-1β, IL-18, and GSDMD gene expressions. These results suggest the efficacy of BMP-7 in attenuating lipid deposition-mediated inflammation-induced pyroptosis and inflammasome formation. 

### 3.8. Effects of BMP-7 Treatment on Cell Signaling Markers TGF-β1, Smad2, and Smad3

**Figure 6:**
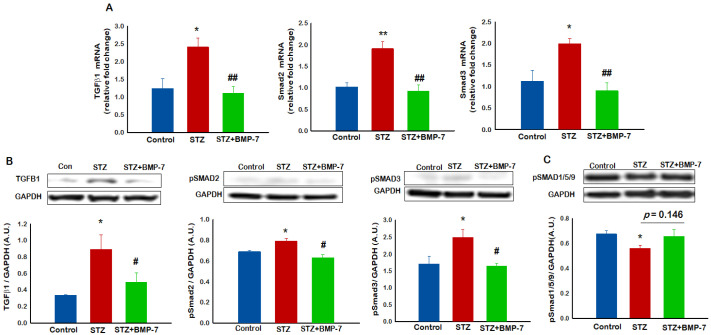
Effects of BMP-7 treatment on cell signaling markers TGF-β1, Smad2, and Smad3. At Day 14 after STZ administration, mice were evaluated for signaling markers TGF-β1, Smad2, and Smad3 by RT-PCR (**A**) and WB (**B**,**C**). As shown in the figure, TGF-β1, Smad2, and Smad3 significantly increased in STZ mice as compared with the control mice. BMP-7-treated diabetic mice had significantly reduced signaling markers. Bar graphs represent: (**A**) Quantitative analysis for relative fold change in gene expressions *n* = 5–6; (**B**,**C**) representative blot and densitometric analysis of signaling markers; (**C**) Western blot densitometric analysis showed a significant decrease in pSMAD1/5/9 in the diabetic group as compared with the control group. An increased trend in pSMAD1/5/9 between the diabetic group versus the BMP-7-treated group was observed, although this increase did not meet statistical significance. Error bars = mean ± standard error of the mean. One-way ANOVA and Tukey tests were performed to assess statistical significance. * *p* < 0.05, ** *p* < 0.001 vs. control; # *p* < 0.05, ## *p* < 0.001 vs. STZ; *n* = 5; *n*, number of animals; TGF-β1, transforming growth factor beta 1; SMAD, suppressor of mothers against decapentaplegic family member.

To examine the involvement of the TGF-β/Smad cell signaling pathway in inflammation-mediated pyroptosis, we used RT-PCR and Western blotting methods to confirm the role of specific markers TGF-β1, Smad2, and Smad3. Our data showed that, in diabetic mice, gene expressions of TGF-β1, Smad2, and Smad3 (Figure 6A,B) were significantly (*p* < 0.05) increased as compared with the control mice, suggesting a role of the TGF-β/Smad cell signaling pathway in hypercholesterolemia-induced pyroptosis and diabetic progression. Furthermore, we observed that, following BMP-7 treatment, gene expressions of TGF-β1, Smad2, and Smad3 (Figure 6A) were significantly (*p* < 0.05) reduced as compared with the diabetic group. Additionally, we performed a Western blot analysis that revealed a significant increase in TGF-β1, pSmad2, and pSmad3 (Figure 6B) protein expressions in diabetic mice as compared with control mice. A significant decrease in these cell signaling markers were observed following BMP-7 treatment, suggesting the efficacy of BMP-7 in attenuating hypercholesterolemia-induced TGF-β1, Smad2, and Smad3 proteins. Further, we also performed a Western blot analysis for downstream markers pSMAD1/5/9 to evaluate the underlying mechanism of this pathway. Our data showed a significant decrease in pSMAD1/5/9 in the diabetic group as compared with the control group. An increased trend in pSMAD1/5/9 (Figure 6C) between the diabetic versus the BMP-7-treated group was observed, although this increase did not meet statistical significance.

### 3.9. Effects of BMP-7 Treatment on Diabetes-Induced Muscle Atrophy and Muscle Degradation

**Figure 7:**
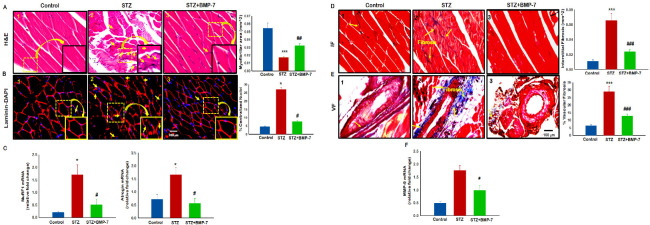
Effects of BMP-7 treatment on Diabetes-induced muscle atrophy, muscle degradation and muscle fibrosis (**A**) Representative photomicrographs (×40) are sections stained with haematoxylin and eosin to detect atrophy (upper panel (**A**) in gastrocnemius muscle on Day-14 after streptozotocin (STZ) administration in control and experimental groups. Stained sections were quantified at ×20 magnification and magnified for visualization of atrophy. Further, IHC staining was performed to determine the muscle deterioration using Laminin and DAPI as shown in lower Panel (**B**). Bar graphs represents quantitative analysis for muscle atrophy and centralized nuclei as indicator of muscle deterioration; (**C**) atrophy gene expressions of MurF1 and atrogin-1, (*n* = 5–8). Scale bar = 100 μm, Error bars = mean ± standard error of the mean. One-way ANOVA and Tukey tests were performed to assess statistical significance. * *p* < 0.05, *** *p* < 0.001 vs. control; # *p* < 0.05, ## *p* < 0.01 vs. STZ *n*: number of animals. MuRF1: muscle ring finger 1 protein. (**D**) Representative images (×40) of Masson’s trichrome staining demonstrated interstitial (upper panel) and vascular fibrosis (lower panel (**E**) in gastrocnemius muscle on Day 14 after STZ administration in control and experimental groups. Stained sections were quantified at ×20 magnification and magnified for visualization of interstitial and vascular fibrosis. Bar graphs represents quantitative analysis for intestinal fibrosis, Percentage of vascular fibrosis quantified over the vessel area, (**F**) MMP-9 gene expression. Error bars = mean ± standard error of the mean. One-way ANOVA and Tukey tests were performed to assess statistical significance. * *p* < 0.05, *** *p* < 0.001 vs. control; # *p* < 0.05, ### *p* < 0.001 vs. STZ. Scale bar = 100 μm; (*n* = 6–8). *n*: number of animals; MMP9: matrix metalloproteinase 9.

Histological staining was performed on GM tissue to determine the effect of BMP-7 on diabetic muscle atrophy. In Figure 7A, representative photomicrographs demonstrated a significant decrease in the GM myocyte area, suggesting muscle atrophy (Figure 7A2) in diabetic mice as compared with the control mice (Figure 7A1). Following treatment with BMP-7, a significant (*p* < 0.05, Figure 7A3) increase in the GM myocyte area was observed, suggesting a decrease in atrophy in diabetic mice. Further, a quantitative analysis confirmed the qualitative data, i.e., the cell size was significantly (*p* < 0.05, Figure 7A) decreased in GM tissues of diabetic mice as compared with the control mice, whereas a significant (*p* < 0.05) increase in muscle cell size was observed with BMP-7 treatment.

Laminin staining was performed to evaluate the presence of muscle degeneration and elevated centralized nuclei. Representative photomicrographs demonstrated a significant increase in laminin accumulation, discontinuous laminin, and centralized nuclei, suggesting muscle degeneration (Figure 7B2) in diabetic mice as compared with the control mice (Figure 7B1). Following treatment with BMP-7, significant (*p* < 0.05, Figure 7B3) decreases in laminin accumulation and centralized nuclei were observed, suggesting a reversal of the diabetic muscle degeneration. Further, quantitative analysis confirmed that the percentage of centrally nucleated muscle fibers was significantly elevated in the diabetic mice GM (Figure 7B 25 ± 5%, *p* < 0.001) as compared with the control mice (4.75 ± 0.5%). A significant reduction (*p* < 0.05) in the centralized nuclei was observed upon BMP-7 treatment. These results suggest that BMP-7 can attenuate muscle degeneration. To confirm attenuated atrophy by BMP-7 at the molecular level, we performed an RT-PCR analysis for MuRF1 and atrogin-1 that have been considered to be major atrophy genes in skeletal muscle [36]. In diabetic mice, both MuRF1 and atrogin-1 gene expressions were significantly (*p* < 0.05, Figure 7C) increased as compared with the control mice, whereas BMP-7 treatment significantly (*p* < 0.05) reduced diabetes-induced MuRF1 and atrogin-1 gene expressions. These data suggest muscle atrophy in diabetes can be attenuated with BMP-7 treatment.

### 3.10. Effects of BMP-7 Treatment on Diabetic Muscle Fibrosis

Skeletal muscle fibrosis is an adverse remodeling mechanism that occurs in diabetes [20]. To determine whether BMP-7 attenuates interstitial and vascular fibrosis in GM muscle of diabetic mice, Masson’s trichrome staining was performed to quantify the presence of collagen. The blue area in representative photomicrographs (Figure 7D) demonstrated significantly (*p* < 0.05) increased interstitial (Figure 7D2) and vascular fibrosis (Figure 7E2) in diabetic mice as compared with the control mice (Figure 7D1,E1). Following BMP-7 treatment, a significant (*p* < 0.05, Figure 7D3,E3) reduction in interstitial and vascular fibrosis was observed. Furthermore, our quantitative IF and VF data were significantly increased (*p* < 0.05, Figure 7B) in diabetic mice as compared with the control mice, whereas BMP-7 treatment significantly (*p* < 0.05) reduced IF and VF in the diabetic mice GM. These results suggest that reduction in collagen deposition and fibrosis further potentiates the therapeutic efficacy of BMP-7 in diabetic animals. To confirm reduced fibrosis by BMP-7, we performed an RT-PCR analysis for MMP9 gene expression. In diabetic mice, MMP9 expression was significantly (*p* < 0.05, Figure 7F) increased as compared with the control mice, whereas BMP-7 treatment significantly (*p* < 0.05) reduced diabetes-induced MMP9 gene expression.

### 3.11. Effects of BMP-7 Treatment on Diabetes-Induced Sarcopenia

**Figure 8:**
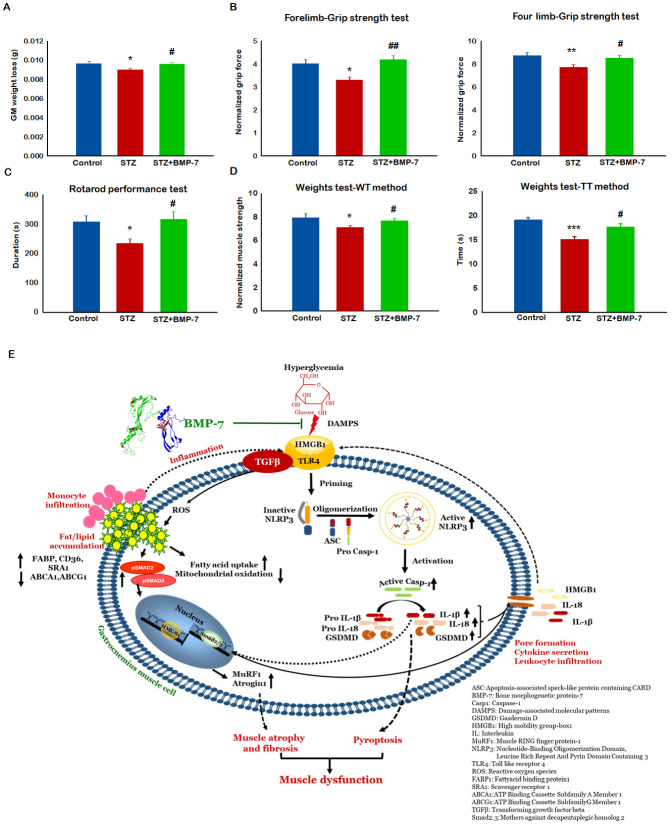
Effects of BMP-7 Treatment on diabetes-induced sarcopenia and muscle dysfunction: (**A**) BMP-7 treatment improves muscle weight and muscle function under hyperglycemic and hyperlipidemic conditions. To evaluate this on Day 14 after streptozotocin (STZ) administration, animals were subjected to different muscle function tests. Bar graphs represent the change in GM weight loss: (**B**) Quantification and analysis for forelimb grip strength, (*n* = 14–16) and four-limb grip strength (*n* = 14–15); (**C**) rotarod function test (*n* = 12–14); (**D**) weights test-WT method *n* = 12–14) and weights test-TT method (*n* = 13–16). Error bars = mean ± standard error of the mean. One-way ANOVA and Tukey tests were performed to assess statistical significance. * *p* < 0.05, ** *p* < 0.01, *** *p* < 0.001 vs. control; # *p* < 0.05, ## *p* < 0.01 vs. STZ; *n*, number of animals; (**E**) schematic representation of overall study. Sarcopenia and adverse muscle remodeling is attenuated by BMP-7 in diabetic mice via the alleviation of lipids, inflammation, HMGB1, and pyroptosis.

To determine the impact of BMP-7 on GM muscle mass after STZ administration, the ratio of muscle weight to body weight was calculated. Our data showed a significantly (*p* < 0.05, Figure 8A) developed sarcopenia (decrease in GM mass) in mice following STZ administration as compared with the control mice. A significant improvement in sarcopenia was observed upon BMP-7 treatment (*p* < 0.05, Figure 8A).

### 3.12. Effects of BMP-7 Treatment on Diabetes-Induced Muscle Dysfunction

To assess whether BMP-7 treatment could improve muscle function, animals were subjected to three different muscle function tests: (1) grip strength for forelimb and four limbs, (2) rotarod test, and (3) weights test for forelimb muscle strength. The grip strength analysis for both forelimbs and combined (four limbs) showed a significant (*p* < 0.05, Figure 8B) deficit in grip strength in diabetic mice as compared with the control mice, whereas BMP-7 treatment significantly (*p* < 0.05) improved grip strength. In the rotarod test, a significant (*p* < 0.05, Figure 8C) decrease in latency to fall was observed in diabetic mice as compared with the control mice. On the other hand, a significant (*p* < 0.05) improvement in diabetic muscle function was observed with BMP-7 treatment, suggesting the potential impact of BMP-7 treatment on muscle dysfunction. For the weights test, a significant (*p* < 0.05) decrease in forelimb muscle strength was observed in diabetic mice as compared with the control mice (Figure 8D) using Weight × Trial and Trial × Time methods. BMP-7 treatment significantly (*p* < 0.05) improved the forelimb muscle strength. These results confirm BMP-7 significantly counteracts diabetes-induced sarcopenia and muscle dysfunction.

## 4. Discussion

Hyperglycemia, oxidative stress, and inflammation are key players in skeletal muscle atrophy, sarcopenia, and adverse remodeling during aging and disease, including cancer and diabetes [4,16,37]. There are numerous treatments available to reduce hyperglycemia-mediated oxidative stress and inflammation [38,39]; however, the development of muscle atrophy, sarcopenia, and adverse muscle remodeling is still progressing and is a major cause of decreased quality of life and high mortality in diabetes. Recent data suggest metabolic alterations in diabetes are a growing concern; therefore, the present study was undertaken to understand whether increased hyperlipidemia could be a major player and causative agent in increasing inflammation, enhanced infiltration of monocytes, pyroptosis leading to fibrosis, atrophy, sarcopenia, and muscle remodeling in diabetes. Furthermore, the present study established the detailed presence of various proinflammatory and anti-inflammatory cytokines, and cellular mechanisms leading to sarcopenia and muscle remodeling. Finally, our data determined the preventive effects of BMP-7, an osteogenic protein, on hyperlipidemia-induced inflammation, cellular mechanisms, sarcopenia, fibrosis, and muscle dysfunction. 

In the present study, our data showed significant increases in hyperglycemia and weight loss which were consistent with previously published reports [4,11,20]. Interestingly, our data showed significantly increased systemic hyperlipidemia in diabetic animals which was consistent with reports of dyslipidemia in diabetic patients [40]. Our data showed increased cholesterol uptake genes (FABP1, CD36, and SRA1) in diabetes, which was in an agreement with recently published studies [41,42]. Moreover, our data showed a significant increase in cholesterol loading genes and reduced RCT genes in diabetic muscle, suggesting the presence of hyperlipidemia at the systemic and molecular level that could imbalance the cholesterol homeostasis in muscle cells. Our data were consistent with previous reports that have suggested specific deletion of ABCA1 and ABCG1 in skeletal muscle reduced muscle mass and function, further increased body fat, and altered glucose metabolism in an age-dependent manner [43]. We also found key metabolism markers PDK4 and CPT1 that were reduced in the diabetic group, which further emphasized metabolic alterations in fatty acid uptake and decreased mitochondrial fatty acid oxidation [41]. Increased hyperlipidemia-specific genes further increased stress-induced myokine FGF21 which was indicative of imbalanced muscle cell functioning [41]. 

However, the present data on hyperlipidemia in diabetes raise a question as to whether increased systemic hyperlipidemia can infiltrate into the muscle tissue, alter cellular homeostasis, and enhance inflammation. We performed lipid-specific perilipin staining on muscle cells to confirm the increased presence of muscle lipids in diabetic mice. Further, we confirmed our data with RT-PCR, suggesting increased infiltrated lipids in the tissue. To the best of our knowledge, this is the first report suggesting increased systemic hyperlipidemia can lead to increased lipid accumulation in muscle tissue. Next, we also performed staining for monocyte infiltration to confirm whether increased muscle tissue lipid levels enhanced monocyte infiltration at the site of increased lipids, which led to inflammation. 

Increased inflammation in diabetic patients and animals has been previously reported [44]. The underlying reason for the initiation and formation of inflammation is not yet known. In this study, we postulate that hyperlipidemia plays a major role in the infiltration of monocytes at the site of lipid-loaded muscle cells which initiates inflammation and inflammation-induced damage-associated molecular pattern (DAMP) cell signaling cell death via pyroptosis. For this purpose, first, we confirmed a detailed analysis of proinflammatory and anti-inflammatory cytokines, followed by their cellular consequences. Our cytokine array data showed an increase in several proinflammatory cytokines that were involved in inducing inflammation (IL-1α, IL-6, MCP1, MIG, TPO, SCF, MCP-1, MCP-5, IFN gamma, leptin, leptin R, IL-17A, RANTES etc.), apoptosis (TNFα, Fas ligand, TNFR1, and TNFRII), pyroptosis (IL-1β), angiogenesis (VEGF), macrophage inflammatory proteins (MIP1α, MIP1γ, MIP3α, and MIP3β), growth factors (M-CSF, GM-CSF, and G-CSF), cell adhesion molecules (VCAM1 and L-selectin), as well as anti-inflammatory cytokines (IL-4, IL-10, and IL-13). Increased anti-inflammatory cytokines under diabetic conditions might suggest the defensive action of the immune system; however, the exact reason for this increase is not well understood. These data were consistent with various previous reports that have suggested an increase in proinflammatory cytokines under diabetic conditions [45,46]. 

Increased hyperlipidemia-induced inflammation stimulates the extracellular release of the nuclear HMGB1, considered to be a DAMP. HMGB1 is present in vascular smooth muscle cells, endothelial cells, and monocytes during homeostasis. The association of HMGB1 with inflammation has been well established, specifically in in vivo mice models [11] and humans suffering from trauma, ischemia, and inflammatory diseases [47]. Consistent with these and our previous studies, HMGB1 was significantly increased in diabetic skeletal muscle cells [11]. In the present study, we reported that increased hyperlipidemia was associated with increased DAMP factor HMGB1 and inflammation in diabetic muscle myopathy, which could be a triggering agent in increased pyroptosis in diabetes. Therefore, we examined downstream factors of HMGB1, such as TLR4, which trigger sterile inflammation as well as form the NLRP3-associated inflammasome and perform pyroptotic cell death. Our data confirmed that increased hyperlipidemia-mediated inflammation activated HMGB1-TLR4 signaling, NLRP3 inflammasome formation, and upregulation of pyroptosis markers caspase-1, IL-1β, IL-18, and the pyroptosis executor GSDMD. These changes indicated pore formation, release of inflammatory cytokines, and cell death through pyroptosis in our diabetic mouse model. In the present study, we observed a significant increase in inflammasome and pyroptosis markers in diabetes, which was consistent with various diabetic cardiovascular, myocardial infarction, and muscle studies [11,12,48]. 

Next, to understand cell signaling mechanisms, we examined the TGF-β/SMAD signaling pathway. Our data showed a significant increase in TGF-β1 with further upregulation of phosphorylated SMAD2 and SMAD3 in diabetes, which was indicative of increased inflammation. Interestingly, our phosphorylated pSMAD1/5/9, which is suggestive of an anti-inflammatory pathway regulator, was decreased in diabetes, emphasizing an enhanced inflammatory microenvironment in the diabetic muscle. Our data were consistent with previously published studies that have shown increased TGF-β and downstream SMAD, suggestive of tissue damage and inflammation [49,50]. A recent study showed diabetes-induced cardiomyopathy mediated via TGF-β and SMAD pathways [49]. Furthermore, TGF-β is a well-known regulator of inflammation, atrophy, fibrosis, and muscle remodeling in skeletal muscle and cardiovascular diseases [51]. 

Therefore, we examined the presence of fibrosis, atrophy, weight loss, and their functional significance in diabetes. We observed that hyperlipidemia-induced pyroptosis cell death in diabetic muscle cells led to significant loss of muscle mass, skeletal muscle atrophy, and fibrosis that contributed to the development and progression of sarcopenia in diabetes. Noticeably, we observed a significant decrease in the loss of myofibrillar area and increased levels of MuRF1 and atrogin1 genes in diabetic mice, suggesting the presence of muscle atrophy. Next, MMP-9 has been considered to be a potential marker for extracellular matrix (ECM) degradation and its association with fibrosis. In our diabetic model, MMP-9 expression was significantly increased. These data were consistent with previous studies that have reported apoptosis and pyroptosis could lead to muscle remodeling in the heart and skeletal muscle [11,12]. 

Moreover, as previously mentioned, hyperlipidemia-induced pyroptosis leading to sarcopenia, atrophy, and muscle dysfunction are major contributors in the development and progression of diabetic muscle myopathy. Therefore, targeting hyperlipidemia can attenuate pyroptosis, which further reduces a series of pathological events, and might be a potential therapeutic agent to treat diabetic muscle myopathy. BMP-7, a commonly used drug to treat osteoporosis in patients, has been tested in animal models of atherosclerosis and found to have anti-inflammatory and anti-fibrotic properties in the heart, muscle, and kidney [52]. The finding of the present study that BMP-7 can reduce systemic hyperlipidemia, infiltration of monocytes, inflammation, and pyroptosis leading to a decrease in sarcopenia, fibrosis, atrophy, and muscle dysfunction in diabetic muscle myopathy is novel. Our BMP-7 data showed a significant decrease in hyperglycemia [11,20], systemic hyperlipidemia, and cholesterol uptake genes. Noticeably, BMP-7 also improved impaired fatty acid oxidation specific genes CPT1 and PDK4, suggesting its regulation in cholesterol metabolism. This improved cholesterol homeostasis with BMP-7 also showed decreased levels of tissue lipids and infiltrated monocytes Furthermore, decreased infiltrated monocytes were also associated with reduced inflammation. BMP-7 treatment reduced many proinflammatory cytokines (almost ranging from 5% to 80%) involved in apoptosis, angiogenesis, immune regulation, macrophage inflammatory proteins, and increased anti-inflammatory cytokines. This information represents a new finding on the wider potential of BMP-7 as an anti-inflammatory agent. Next, our data confirm that BMP-7 reduces levels of pyroptosis initiator, inflammasome formation, and pyroptosis. BMP-7 inhibited the inflammatory TGF-β1/SMAD2/3 cell signaling pathway, whereas it enhanced phosphorylated SMAD1/5/9 cell signaling protein, aiding in the regulation and maintenance of muscle mass. This BMP-7 data suggest a role in inhibitory cell signaling of muscle loss and wasting.

In this study, muscle fibrosis data showed a significant reduction in collagen deposition following BMP-7 treatment. Additionally, the expression level of MMP-9 reduced significantly following BMP-7 treatment, which suggests BMP-7 treatment decreased both vascular and interstitial fibrosis as well as extracellular profibrotic protein MMP-9 in the diabetes-induced muscle myopathy. These data were in agreement with previously published reports [11,12] on diseases of the kidney, heart, and muscle. Interestingly, the present study data show that BMP-7 treatment significantly reduces hyperlipidemia-induced pyroptosis-associated muscle atrophy and sarcopenia, while improving diabetic muscle dysfunction, indicating BMP-7 could serve as a future therapeutic agent to treat diabetic muscle myopathy. Insulin resistance plays a role in muscle damage. However, the current study did not explore the role of insulin with and without BMP-7 treatment. Therefore, future studies are suggested to understand the role of insulin following BMP-7 treatment. 

## 5. Conclusions

In conclusion, we report, for the first time, that hyperlipidemia could be among the major causative agents in diabetes-induced monocyte infiltration and tissue inflammation. Furthermore, our data show a flurry of cytokine upregulation that creates an imbalance in DAMP protein(s) such as HMGB1, which further regulate TLR4-NLRP3 inflammasome formation leading to inflammation and cell death through pyroptosis. Altered cholesterol homeostasis and inflammation led to a defective TGF-β1/SMAD2/3 signaling pathway, inducing fibrosis, atrophy, muscle remodeling, and sarcopenia (Figure 8E). Evident amelioration of metabolic gene expression, increased inflammation and pyroptosis, increased pathological cell signaling, sarcopenia, and decreased muscle function data show that treatment with BMP-7 is promising. Moreover, our data suggest this agent, which has never been tested for hyperlipidemia-induced inflammation-mediated sarcopenia in young mice, could be a future therapeutic agent to treat diabetic myopathy and to improve quality of life.

## Data Availability

Data are contained within the article and Supplementary Materials.

## Supplementary Materials

The following supporting information can be downloaded at: https://www.mdpi.com/article/10.3390/antiox12020331/s1, Figure S1: Effect of BMP-7 on M1 polarization, Table S1: Primers and antibodies used in the study. References [11,12,25,27,53,54,55,56,57,58,59] are cited in the supplementary materials.
