# Supplementary material for: BMP-7 Attenuates Sarcopenia and Adverse Muscle Remodeling in Diabetic Mice via Alleviation of Lipids, Inflammation, HMGB1, and Pyroptosis
Source: Antioxidants (Basel). 2023 Jan 31;12(2):331. doi: 10.3390/antiox12020331 (PMC9952667; doi:10.3390/antiox12020331)
Supplement: Supplementary file 1 [file antioxidants-12-00331-s001.zip › antioxidants-2186456-supplementary.pdf]

BMP-7 attenuates Sarcopenia and Adverse Muscle Remodeling in Diabetic Mice via Alleviation of Lipids, Inflammation, HMGB1, and Pyroptosis

Chandrakala Aluganti Narasimhulu and Dinender K. Singla *

Division of Metabolic and Cardiovascular Sciences, Burnett School of Biomedical Sciences, College of Medicine, University of Central Florida, Orlando, FL 32816, USA

* Correspondence: dinender.singla@ucf.edu; Tel.: +1-407-823-0953; Fax: 407-823-0956

Abbreviations

ABCA1: ATP binding cassette subfamily A member 1; ABCG1: ATP binding cassette subfamily G member 1; ANOVA: Analysis of variance; ASC: Apoptosis-associated speck-like protein containing a c-terminal caspase recruitment domain; A.U: Arbitrary units; BMP-7: Bone morphogenetic protein-7; BW: Body weight; DAPI: 4', 6-diamino-2-phenylindole; CPT1: Carnitine palmitoyltransferase 1; DAMPS: Damage associated molecular patterns; FABP1: Fatty acid binding protein 1; FasL: Fas ligand; GAPDH: FGF21: Fibroblast growth factor 21; GAPDH: Glyceraldehyde 3-phosphate dehydrogenase; GCSF: Granulocyte colony-stimulating factor; GM: Gastrocnemius muscle; GM-CSF: Granulocyte-macrophage colony-stimulating factor; GS: Goat serum; GSDMD: Gasdermin D; GTT: Glucose tolerance test; HDL: High-density lipoprotein; HMGB1:High mobility group box1; IHC: Immunohistochemistry; IGFBP-5:insuln-like growth factor binding protein 5; IF: Interstitial fibrosis; IL: Interleukin; iNOS: Inducible nitric oxide synthase; KC: Keratinocyte chemoattractant; LDL: Low-density lipoprotein; M-CSF: macrophage colony-stimulating factor; MIP: Macrophage inflammatory protein; MIG: Monokine induced gamma interferon; MuRF1: Muscle RING-finger protein-1; MMP9: matrix metalloproteinase 9; NLRP3: Nucleotide-Binding Oligomerization Domain, Leucine Rich Repeat And Pyrin Domain Containing protein 3; PDK4: Pyruvate dehydrogenase kinase 4; SR-A1: Scavenger receptor A1; STZ: Streptozotocin; TLR: Toll-like receptor; IP: Intraperitoneal; IV: Intravenous PBS: Phosphate

buffered saline; PCR: Polymerase chain reaction; PF4: Platelet factor 4; PVDF: Polyvinylidene difluoride; RANTES: Regulated upon activation, normal T cell expressed; RIPA: Radioimmunoprecipitation assay; SCF: stem cell factor; SDS: Sodium dodecyl sulfate; SEM: Standard error of mean; SMAD: Suppressor of Mothers Against Decapentaplegic family member; TBS: Tris buffer saline; TGFβ1: Transforming growth factor beta1; TNFRII: Tumor necrosis factor receptor II; TPO: Thyroperoxidase; TRG: Triglycerides; VCAM1: Vascular cell adhesion molecule 1; VEGF: Vascular endothelial growth factor; VF: Vascular fibrosis; VLDL: Very low density lipoprotein.

Figure S1.

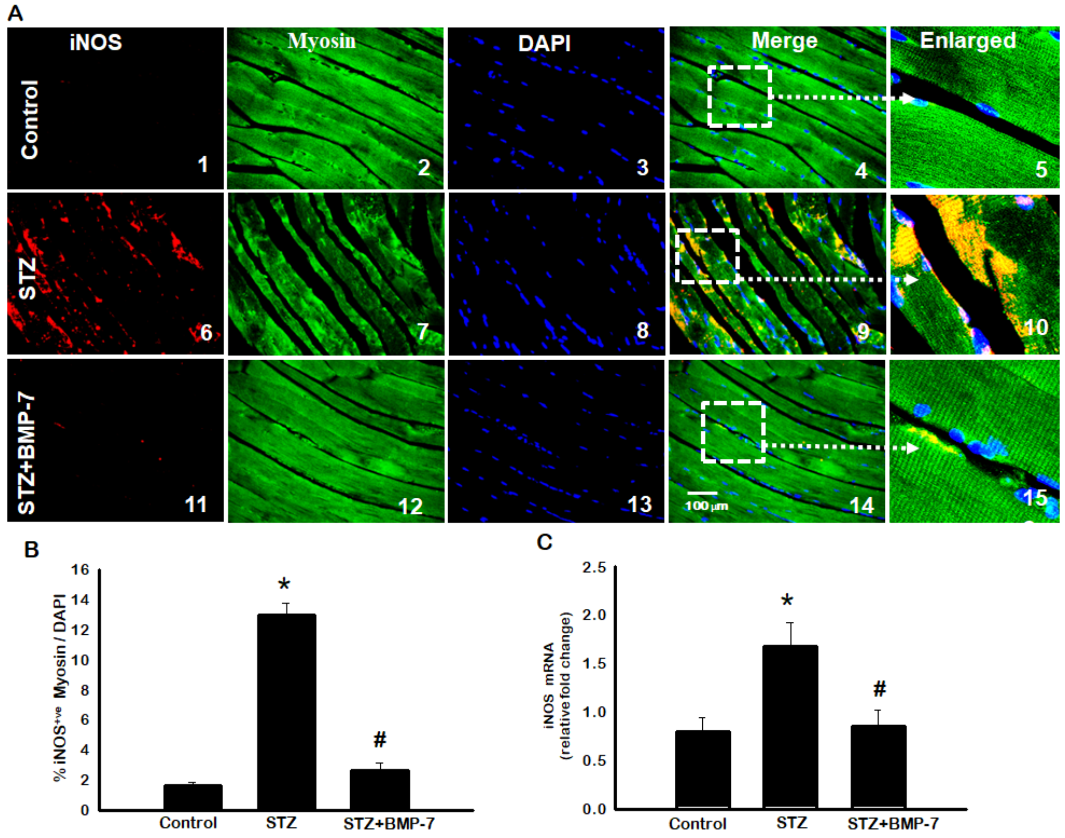

Figure S1. Effects of BMP-7 treatment on M1 macrophage polarization.

Hyperlipidemia-induced oxidative stress causes monocyte infiltration and M1 macrophage formation which further cellular pathological mechanisms. Representative images of IHC staining

for (**A**) iNOS expression at D14 shows that administration of STZ significantly increases iNOS expression in STZ mice (6~10) as compared to control (1-5). BMP-7 treated diabetes mice had significantly lower number of +ve cells (11-15). Panel A in all figures showed iNOS markers in red (1, 6, 11), muscle cells in green (2, 7, 12j), DAPI in blue (3, 8, 13), and merged images (4, 9, 14). Scale bar = 100μm. White boxes and arrows indicates enlarged section of merged images (5, 10, 15). Quantitative analysis in bar graph for IHC (n=6-8) (**B**) gene expression (n=5-6) (**C**) shows increased expression of iNOS in GM of STZ mice vs. control mice, whereas BMP-7 treatment showed significant reduction iNOS in diabetic mice. * $p < 0.05$ vs. control; # $p < 0.05$ vs STZ.

Table S1. List of Antibodies and primers used for the study.

A: List of antibodies

Primary antibodies

Antibody	Dilution for IHC	Dilution for WB	Supplier and cat no.	References
Myosin	1:50		Sigma (Cat. #M7523)	[1]
CD14	1:100		Abbiotec (Cat. #251561)	[2]
iNOS	1:100		Abcam (Cat. #ab15323)	[2,3]
HMGB1	1:600		Abcam (Cat. #ab79823)	[1]
TLR-4	1:50		Abcam (Cat. #ab13556)	[1,3]
NLRP3	1:50		Abcam (Cat. #ab98151)	[1,3]
Caspase1	1:50		Abcam (Cat. #ab1872)	[1,3]
IL-1β	1:50		Abcam (Cat. #ab9722)	[1,3]
IL-18	1:50		Abcam (Cat. #ab71495)	[1,3]
GSDMD	1:600		Abcam (Cat. #ab219800 and ab209845)	[1,3]
Perilipin	1:200		Abcam (Cat. #ab3526)	[4]
Laminin	1:200		Sigma (Cat. #L9393)	[4]
TGF-β1		1:1000	Abcam (Cat. #92486)	[5,6]
P-SMAD2		1:1000	Cell signaling (Cat. # 18338S)	[7,8]
P-SMAD3		1:1000	Cell signaling (Cat. # 9520S)	[8]
P-SMAD1/5/9		1:1000	Cell signaling (Cat. # 13820S)	[9,10]
GAPDH		1:1000	Cell signaling (Cat. #5174)	[11]

Secondary antibodies

Antibody	Dilution for IHC	Dilution for WB	Supplier and cat no.	
Alexa Fluor 488 goat anti-rabbit	1:50		Invitrogen (Cat. #A11008)	[1,3]
Alexa Fluor 568 goat anti-rabbit	1:50		Invitrogen (Cat. #A11011)	[1,3]
IgG HRP		1:1000	Cell signaling (Cat. #7074S)	[1,3]

B. Mouse-primer sequences

Target	Gene Accession Nos	Forward Primer	Reverse Primer
ABCA1	AF287263	5'-GGGAATTGAACCTGAGTCCT-3'	5'-AGTCATTCCTCCTCCCATTC-3'
ABCG1	AF323659	5'-CCAGACAGTTGTGGATGTGG-3'	5'-GACCTCGCTCTTCCTTCCTT-3'
Atrogin1	AF441120	5'-CCATCAGGAGAAGTGGATCTATGTT-3'	5'-GCTTCCCCCAAAGTGCAGTA-3'
GAPDH	GU214026	5'-ACCCAGAAGACTGTGGATGG-3'	5'-CACATTGGGGGTAGGAACAC-3'
Caspase-1	NM_009807	5'-GAAACGCCATGGCTGACAAG-3'	5'-CGTGCCTTGTCCATAGCAGT-3'
CD14	NM_009841	5'-TCAGGAACTCTGGCTTTGCT-3'	5'-TGGCTTTTACCCACTGAACC-3'
CD36	NM_001159555	5'-TGCTGGAGCTGTTATTGGTG-3'	5'-TGGGTTTTGCACATCAAAGA-3'
CPT1	NM_009948	5'-CATGTATCGCCGCAAACTGG-3'	5'-CCTGGGATGCGTGTAGTGTT-3'
FABP1	NM_017399	5'-GGAAGGACATCAAGGGGGTG-3'	5'-TCACCTTCCAGCTTGACGAC-3'
FGF21	NM_020013	5'-GCCTTGAAGCCAGGGGTCT-3'	5'-GCTGCAGGCCTCAGGATA-3'
GSDMD	NM_026960	5'-GACTCTGGAGAACTGGTGCC-3'	5'-ACACAGAACTCTGCTCCTGC-3'
HMGB1	NM_010439	5'-CGCTGGCTGGAGAGTAATGT-3'	5'-AGGATCTCCTTTGCCCATGT-3'
IL-1β	NM_008361	5'-AACCTGCTGGTGTGTGACTTC-3'	5'-CAGCACGAGGCTTTTTTGT-3'
IL-18	NM_008360	5'-ACTTTGGCCGACTTCACTGT-3'	5'-GTCTGGTCTGGGGTTCACTG-3'
iNOS	NM_010927	5'-CGAAACGCTTCACTTCCAA-3'	5'-TGAGCCTATATTGCTGTGGCT-3'
MMP-9	NM_013599	5'- CGACGACGACGAGTTGTG-3'	5'-CTGTGGTGCAGGCCGAATAG -3'
MuRF1	NM_001039048	5'-AGGGAGCAGCTGGAAAAGTC-3'	5'-TCCCAAAGTCAATGGCCCTC-3'
NLRP3	NM_145827	5'-CACGAGTCCTGGTGACTTTGTA-3'	5'-CAGCCCTTTCGAGGGTCTC-3'
Perilipin	NM_175640	5'-AGCGTGGAGAGTAAGGATGTC-3'	5'-CTTCTGGAAGCACTCACAGG-3'
PDK4	AF239176	5'-AATGCCCAGGCCCACTTAAC-3'	5'-TGAGGACCGCCTTTAGTTGA-3'
TLR-4	AF185285	5'-TTCCTTCTTCAACCAAGAACATAGATC-3'	5'-TTGTTTCAATTTCACACCGGATAA-3'
TGF-β1	M13177	5'-TGAGTGGCTGTCTTTTGACG-3'	5′-GGTTCATGTCATGGATGGTG-3'
SMAD2	NM_001252481	5'-CAGGACGGTTAGATGAGCTTGAGA-3'	5′-CCCACTGATCTACCGTATTTGCTG-3'
SMAD3	NM_016769	5′-AACGTGAACACCAAGTGCA-3'	5′-ACAGGCGGCAGTAGATAACG-3'
SRA1	NM_001113326	5'-AAAGGTGATCGGGGACAAA-3'	5'-TTGCCCCAATATGATCAGG-3'

References:

1. Aluganti Narasimhulu C, Singla DK. Amelioration of diabetes-induced inflammation mediated pyroptosis, sarcopenia, and adverse muscle remodelling by bone morphogenetic protein-7. Journal of Cachexia, Sarcopenia and Muscle. 2021;12:403-20. doi:https://doi.org/10.1002/jcsm.12662
2. Elmadbouh I, Singla DK. BMP-7 Attenuates Inflammation-Induced Pyroptosis and Improves Cardiac Repair in Diabetic Cardiomyopathy. Cells. 2021;10:doi:10.3390/cells10102640
3. Singla DK, Johnson TA, Tavakoli Dargani Z. Exosome Treatment Enhances Anti-Inflammatory M2 Macrophages and Reduces Inflammation-Induced Pyroptosis in Doxorubicin-Induced Cardiomyopathy. Cells. 2019;8:doi:10.3390/cells8101224
4. Gibbons MC, Singh A, Anakwenze O, Cheng T, Pomerantz M, Schenk S, et al. Histological Evidence of Muscle Degeneration in Advanced Human Rotator Cuff Disease. The Journal of bone and joint surgery American volume. 2017;99:190-9. doi:10.2106/JBJS.16.00335
5. Liu X, Sun Z, Wang H. Metformin alleviates experimental colitis in mice by up-regulating TGF-β signaling. Biotech Histochem. 2021;96:146-52. doi:10.1080/10520295.2020.1776896
6. Wang D, Yin Y, Wang S, Zhao T, Gong F, Zhao Y, et al. FGF1(ΔHBS) prevents diabetic cardiomyopathy by maintaining mitochondrial homeostasis and reducing oxidative stress via AMPK/Nur77 suppression. Signal Transduct Target Ther. 2021;6:133. doi:10.1038/s41392-021-00542-2
7. Dalvand A, da Silva Rosa SC, Ghavami S, Marzban H. Potential role of TGFB and autophagy in early crebellum development. Biochem Biophys Rep. 2022;32:101358. doi:10.1016/j.bbrep.2022.101358
8. Bertrand-Chapel A, Caligaris C, Fenouil T, Savary C, Aires S, Martel S, et al. SMAD2/3 mediate oncogenic effects of TGF-β in the absence of SMAD4. Commun Biol. 2022;5:1068. doi:10.1038/s42003-022-03994-6
9. Xiao Y, Donnelly H, Sprott M, Luo J, Jayawarna V, Lemgruber L, et al. Material-driven fibronectin and vitronectin assembly enhances BMP-2 presentation and osteogenesis. Mater Today Bio. 2022;16:100367. doi:10.1016/j.mtbio.2022.100367
10. Rocher C, Singla DK. SMAD-PI3K-Akt-mTOR pathway mediates BMP-7 polarization of monocytes into M2 macrophages. PloS one. 2013;8:e84009. doi:10.1371/journal.pone.0084009
11. Qiu Q, Yang L, Feng Y, Zhu Z, Li N, Zheng L, et al. HDAC I/IIb selective inhibitor Purinostat Mesylate combined with GLS1 inhibition effectively eliminates CML stem cells. Bioact Mater. 2023;21:483-98. doi:10.1016/j.bioactmat.2022.08.006
